# Room temperature stable film formation of π-conjugated organic molecules on 3*d* magnetic substrate

**DOI:** 10.1038/s41598-017-18605-2

**Published:** 2018-01-10

**Authors:** Eiichi Inami, Mikio Shimasaki, Hideki Yorimitsu, Toyo Kazu Yamada

**Affiliations:** 1 Graduate School of Engineering, Chiba University, 1-33 Yayoi-cho, Inage-ku, Chiba, 263-8522 Japan; 2 Graduate School of Advanced Integration Science, Chiba University, 1-33 Yayoi-cho, Inage-ku, Chiba, 263-8522 Japan; 3 Department of Chemistry, Graduate School of Science, Kyoto University, Kitashirakawa, Oiwakecho, Sakyo-ku, Kyoto, 606-8502 Japan; 4 Molecular Chirality Research Center, Chiba University, 1-33 Yayoi-cho, Inage-ku, Chiba, 263-8522 Japan

## Abstract

An important step toward molecule-based electronics is to realize a robust and well-ordered molecular network at room temperature. To this end, one key challenge is tuning the molecule–substrate electronic interactions that influence not only the molecular selfassembly but also the stability of the resulting structures. In this study, we investigate the film formation of π-conjugated metal-free phthalocyanine molecules on a 3*d*-bcc-Fe(001) whisker substrate at 300 K by using ultra-high-vacuum scanning tunneling microscopy. On bare Fe(001), hybridization between the molecular π and the Fe(001) *d*-states prevents the molecular assembly, resulting in the disordered patchy structures. The second- and third-layer molecules form densely packed films, while the morphologies show clear difference. The second-layer molecules partially form p(5 × 5)-ordered films with the rectangular edges aligned along the [100] and [010] directions, while the edges of the third-layer films are rounded. Remarkably, such film morphologies are stable even at 300 K. These findings suggest that the molecular self-assembly and the resulting morphologies in the second and third layers are affected by the substrate bcc(001), despite that the Fe-*d* states hybridize only with the first-layer molecules. The possible mechanism is discussed with the kinetic Monte Carlo simulation.

## Introduction

Organic molecules grown on crystalline substrates have received remarkable attention as an alternative to inorganic semiconductors because of their relatively low-cost synthesis and flexibility in tailoring their properties^1,2^. Extensive investigation on molecules unveiled exciting novel properties that provide promising applications in a wide variety of areas as high-functional molecule-based devices^3–11^. One key challenge to achieve molecular devices is the sophisticated regulation of the molecular assembly into well-ordered network (molecular film). Furthermore, their practical applications require the stability of the molecular film against thermal fluctuations at room temperature (RT).

Among several organic molecules, a family of phthalocyanines (Pc), which are typical *π*-conjugated molecules, are most extensively studied because of their fascinating properties, such as thermal and chemical stabilities, strong optical response in the visible region, and a wide variety of derivatives depending on the center-coordinated metal atoms. These features support their use in electroluminescent devices^12^, photovoltaic cells^13^, and field effect transistors^14^.

Pc single molecules and their film formations were successively investigated by using scanning tunneling microscopy and spectroscopy (STM/STS). Numerous studies have been reported particularly on the Pc molecules grown on highly oriented pyrolytic graphite (HOPG)^15–17^, and on various noble metals (such as Au, Ag, Cu, and Pt)^18–21^. As these substrate surfaces, which are characterized by *s* or *p* derived states, are chemically inert, the molecules are mostly physisorbed on the substrates through van der Waals interactions. This allows the Pc to thermally diffuse along the surface, resulting in the formation of two-dimensional (2D) ordered films. However, the realization of a robust molecular film at RT is not easy. A few studies reported that, at 300 K, the high-mobility molecules on these substrates destabilize the film morphologies particularly at the edges^15^.

On the other hand, magnetic metals such as Fe, Co, Cr, and Mn have attracted increasing interest as the substrates of *π*-conjugated organic molecules. Magnetic metals are distinct from HOPG and noble metals in which the surface states near the Fermi energy are dominated by the *d* state. Recently, STM studies^22–24^ reported that the interactions between the 3*d* magnets and the *π*-conjugated single molecules (hybridized *π*–*d* states) induce giant magnetoresistance, which is a key feature for achieving spintronics devices, such as efficient data storage and computing devices. Significantly, the hybridized *π*–*d* states lead to strong chemisorption of the adsorbed single molecules on the substrate. For this reason, the molecules are immobile even at RT^25^. This encourages us to utilize the magnetic substrate for the fabrication of robust molecular films.

In this paper, using ultra-high-vacuum (UHV) STM, we have investigated the morphological characteristics of *π*-conjugated metal-free phthalocyanine (H_2_Pc) molecules on magnetic Fe(001) substrate from low coverage to multilayer coverage regions at 300 K. Under monolayer coverages, H_2_Pc molecules on bare Fe(001) exhibited as disordered patchy structures. Above monolayer coverages, the second- and third-layer molecules formed densely-packed films, which morphologies were found to be stable even at 300 K. The morphologies also showed clear difference depending on the layer number. The second-layer films partially showed p(5 × 5)-ordered arrays with the rectangular edges aligned along the [100] and [010] directions, while the third-layer films showed the rounded edges. These findings suggest that the film growth processes and the resulting morphologies in the second and third layers are affected by the substrate bcc(001) despite that the Fe-*d* states hybridize only with the first-layer molecules. We discussed the layer-dependent molecules–substrate interactions, and then proposed possible film growth processes that was reasonably examined by a kinetic Monte Carlo simulation.

## Results and Discussion

### H_2_Pc/Fe(001) in low coverage regions (0.1–0.8 ML)

**Figure 1:**
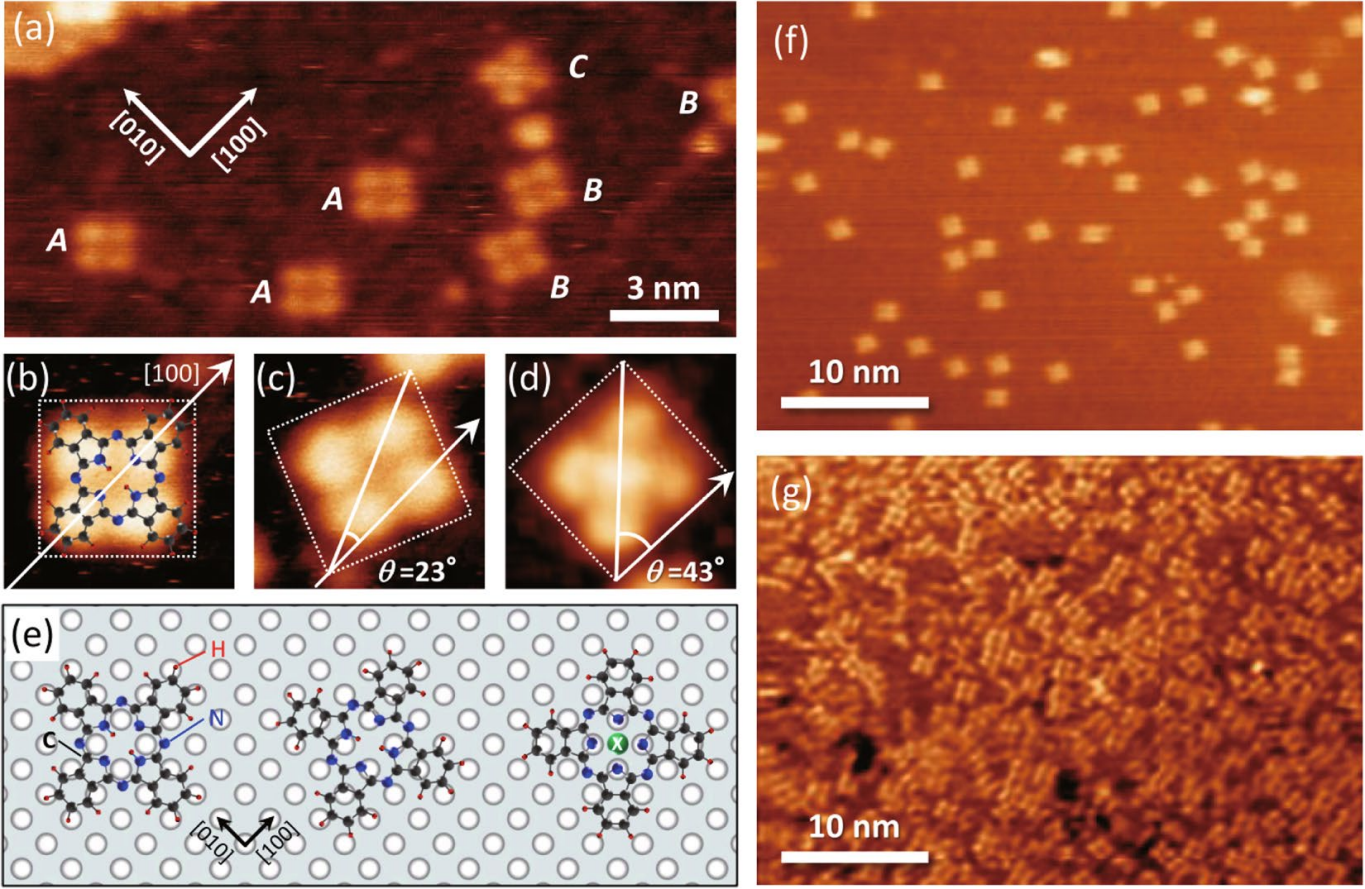
H_2_Pc/Fe(001) in low-coverage regions. (**a**) STM topography image of Fe(001) with 0.1 ML coverage of H_2_Pc molecules (9 nm × 20 nm, *V*_s_ = −1.5 V, *I*_t_ = 300 pA). *A*, *B*, and *C* denote type A, type B, and type C molecules, respectively (see text). (**b**–**d**) Magnified images of type A, (**b**), type B, (**c**), and type C, (**d**), molecules. (**e**) Structural ball-stick models of type A (left), type B (middle), and type C (right) molecules. A green ball labeled *X* indicates unknown-atomic impurity. (**f**,**g**) STM topography images of Fe(001) surfaces (30 nm × 40 nm) with (**f**) 0.1 ML (*V*_s_ = −1.0 V, *I*_t_ = 200 pA), (**g**) 0.8 ML (*V*_s_ = −0.6 V, *I*_t_ = 500 pA) coverage of H_2_Pc molecules.

**Figure 2:**
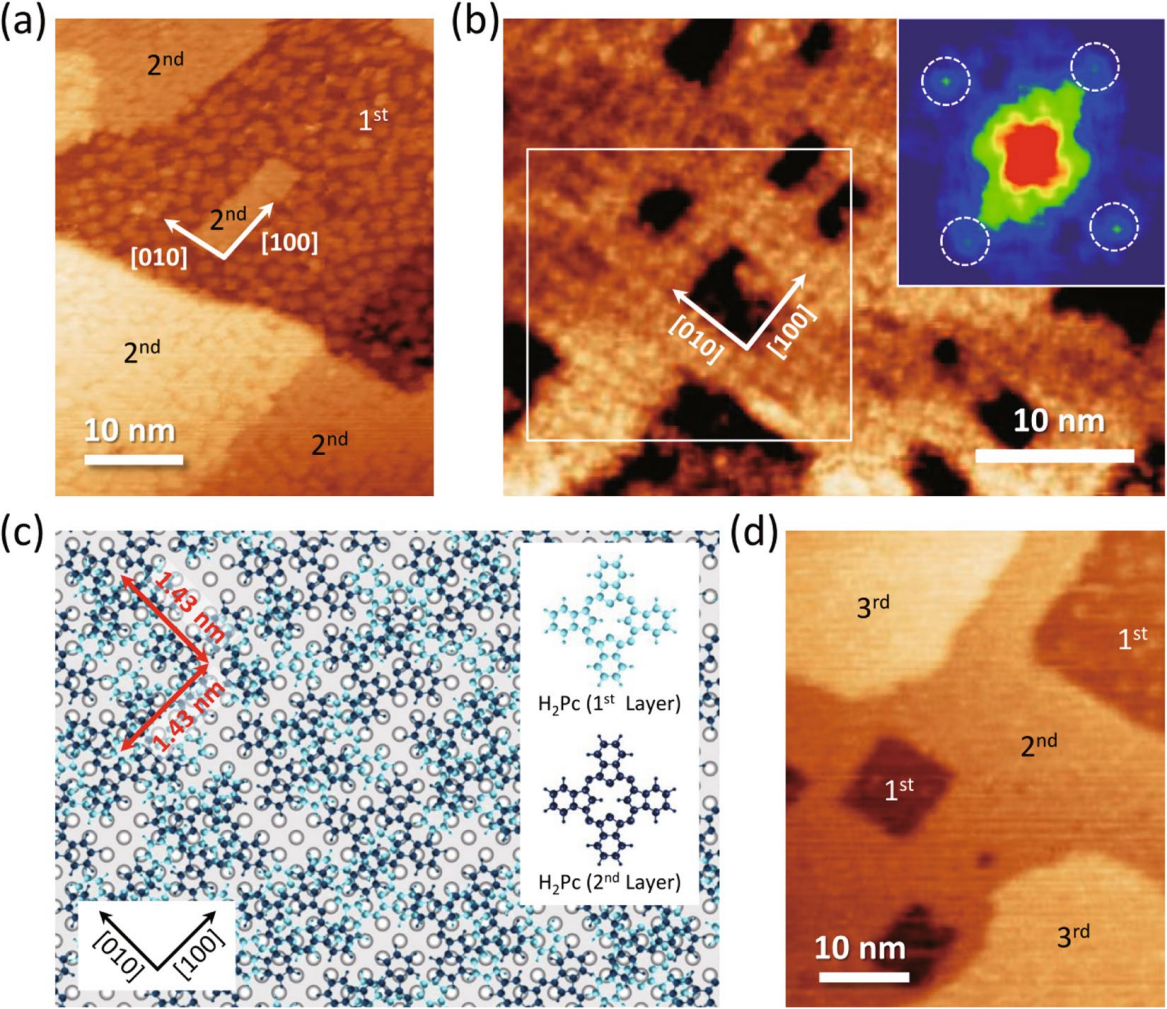
H_2_Pc/Fe(001) in high coverage regions. (**a**) STM topography image (48 nm × 38 nm, *V*_s_ = −0.8 V, *I*_t_ = 100 pA) of Fe(001) surfaces with 1.5 ML coverage of H_2_Pc molecules. (**b**) Molecular resolution STM image of second H_2_Pc layer (30 nm × 40 nm, *V*_s_ = −2.0 V, *I*_t_ = 500 pA). The lateral thermal drift of the image was compensated (see the Method section). Inset image shows 2D fast Fourier transform (2D-FFT) of the rectangular area in (**b**). (**c**) Sketch of the proposed molecular arrangements in first and second layers. The adsorption configuration of the second-layer molecule was assumed to type B. (**d**) STM topography image (53 nm × 42 nm, *V*_s_ = −1.5 V, ${I}_{{\rm{t}}}$ = 50 pA) of Fe(001) surfaces with 2.4 ML coverage of H_2_Pc molecules.

Figure 1(a) and (f) show the STM images of H_2_Pc single molecules on Fe(001) at a coverage of 0.1 ML. One can identify the isolated single molecules with characteristic four-lobed shapes. Each lobe corresponds to one H_2_Pc arm, thereby confirming that the molecular plane is oriented parallel to the substrate surface. Our previous study^25^ revealed that the H_2_Pc single molecules are adsorbed onto the Fe(001) substrate with three in-plane orientations, namely type A, type B, and type C [Fig. 2(e)]. The type A molecule, which exhibits the most energetically stable geometry, is adsorbed parallel to the [100] direction. The type B molecule, which exhibits the second most stable geometry, is adsorbed with a rotation of approximately 23 ± 2 deg. out of the [100] direction. The type C molecule, which is rarely observed and expected to include some impurities at the core, is adsorbed with a rotation of approximately 45 ± 2 deg. out of the [100] direction. In the present experiment, these adsorption geometries were confirmed in the STM images [Fig. 1(b)–(d)].

We increased the coverage of H_2_Pc molecules up to 0.8 ML [Fig. 1(g)]. Although most of the Fe(001) surface was covered with molecules, they did not form domains but still remained isolated, thereby resulting in disordered patchy structures. This result reveals that the molecules absorbed on the Fe(001) surface are immobile, and they cannot aggregate with each other. The reason for this activity is discussed in detail later. It was also found that the molecules did not form second layers. Therefore, under monolayer coverages, the molecules prefer to be adsorbed on the Fe(001) substrate, that is, adsorptions onto the pre-existing molecules are unfavorable.

### H_2_Pc/Fe(001) in high coverage regions (>1 ML)

Above monolayer coverages, H_2_Pc molecules started forming multilayers. Figure 2(a) shows the STM image of approximately 1.5 ML H_2_Pc molecules on Fe(001) substrate. Interestingly, the second-layer molecules (labeled “2^nd^”) were revealed to form films with densely packed structures, while the substrate (first-layer molecules) remained as disordered patchy structures. It is also noteworthy that the edges of the films align along the [100] and [010] directions, which are crystallographically equivalent orientations of the Fe(001) surface. The molecular resolution image of the second layer is shown in Fig. 2(b). The molecules were found to form partially-ordered arrays. From the fast Fourier transform (FFT) analysis of the rectangular region in Fig. 2(b) [inset in Fig. 2(b)], the intermolecular distance in the ordered domain was evaluated to be 1.43 ± 0.02 nm; thus, the molecules form p(5 × 5) superstructure with respect to the Fe(001) surface. Under the assumptions that the adsorption geometries (adsorption site and the in-plane rotational angle) of the film constituent molecules are the same with those in the first layer, the molecular arrangements in the first and second layers were deduced as sketched by Fig. 2(c). In Fig. 2(c), the hollow sites of Fe(001) are partially occupied by the first-layer molecules (colored light blue) with two typical configurations (type A and type B), above which the second-layer molecules (colored dark blue) are adsorbed with type B configuration at the sites corresponding to the hollow sites of Fe(001). For p(5 × 5) structure, the type A configuration (see Supplementary Figure 2) is considered to be energetically unfavorable due to the repulsive H-H interactions between benzene groups of neighboring molecules. We emphasize that, even under the dense-packed ordered second-layer, the first layer should not be packed but still forms disordered patchy structure. That is, the some of the second-layer molecules should not be “perfectly” but “partially” supported by the first layer. This is undetectable by STM, but reasonable based on the fact that the chemisorbed first-layer molecules do not diffuse and immobilize even by the STM manipulation^25^. At a coverage of 2.4 ML [Fig. 2(d)], the molecules in the third layer also formed densely packed structures (labeled “3^rd^”) as well as the growth of the second layers. However, in comparison with the second layer, the edges of the third layers were found to be rounded.

**Figure 3:**
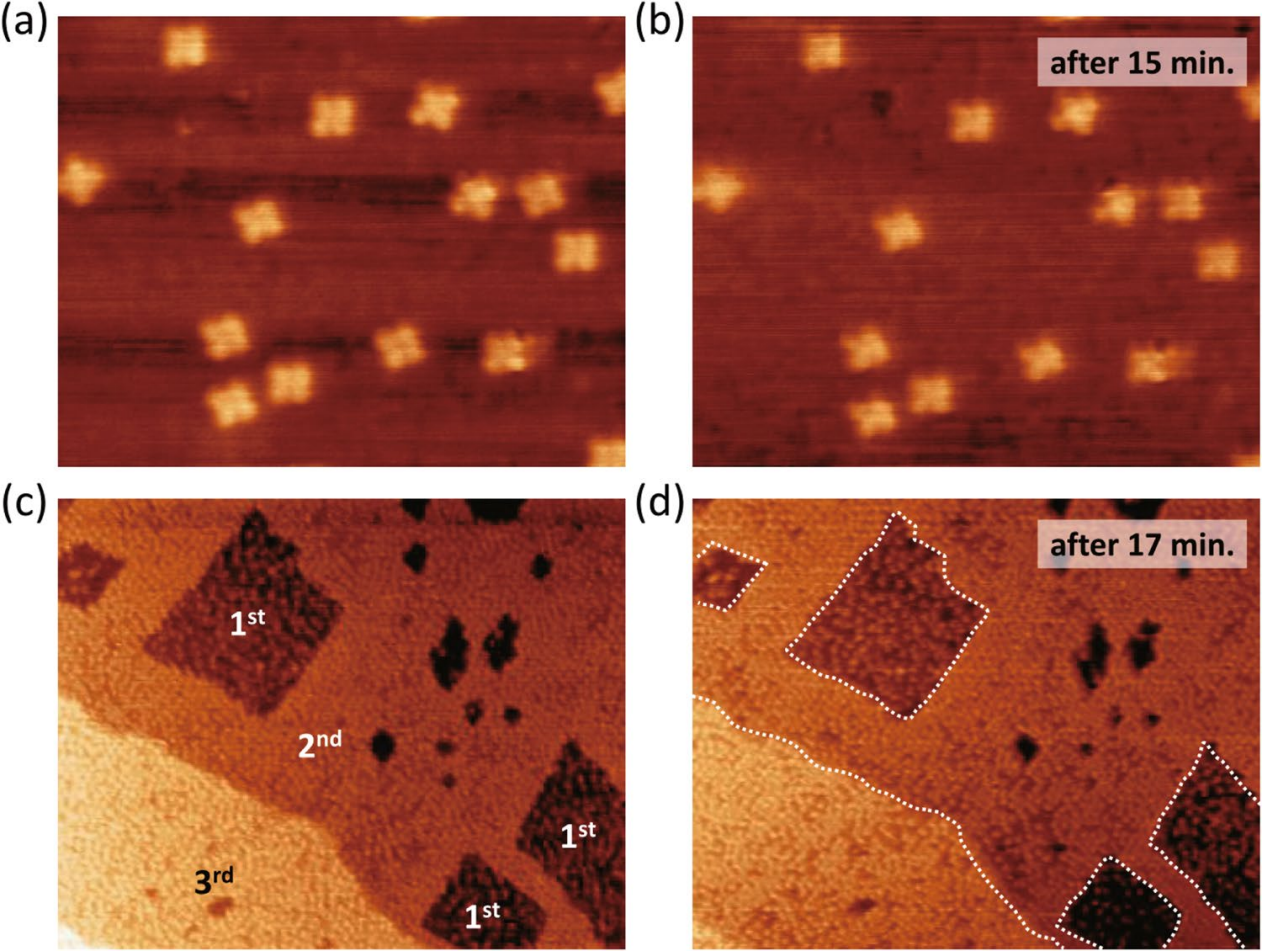
Thermal stability of H_2_Pc on Fe(001). (**a**,**b**) Continuous STM topography images of H_2_Pc molecules on Fe(001) surface (16 nm × 20 nm, *V*_s_ = −1.0 V, *I*_t_ = 200 pA). (**c**,**d**) Continuous STM topography images including first, second, and third H_2_Pc layers on Fe(001) surface (64 nm × 77 nm, *V*_s_ = −2.0 V, *I*_t_ = 500 pA). Labeled numbers, 1^st^, 2^nd^, and 3^rd^, indicate first, second, and third H_2_Pc layers, respectively.

The thermal stability of the H_2_Pc single molecules and the films on Fe(001) were investigated by consecutive STM imaging. Figure 3(a) and (b) show the consecutive STM images focused on the H_2_Pc single molecules on the Fe(001) surface. To check the stability, first, a particular area including single molecules was imaged. Then, the same area was imaged again after 15 min. Since the image area shifts with time due to the lateral thermal drift, the overlap regions of the two images were extracted to investigate the morphological variation at exactly the same area (the detailed procedure is described in Supplementary Method 1). It is clear that no changes in the adsorption sites and the geometries were observed during the repeated scans. This result indicates that the H_2_Pc single molecules on Fe(001) are thermally stable at RT, as reported by the previous study^25^. In similar manner, the consecutive STM images of the H_2_Pc films are also obtained as shown by Fig. 3(c) and (d), where Fig. 3(d) was obtained 17 min after the STM image in Fig. 3(c) was obtained (see Supplementary Method 1). We can observe that the edge features in Fig. 3(c) are maintained by repeated imaging, as indicated by dashed lines in Fig. 3(d). Therefore, the H_2_Pc molecules form densely packed and thermally stable films in the second and the third layers even at RT.

**Figure 4:**
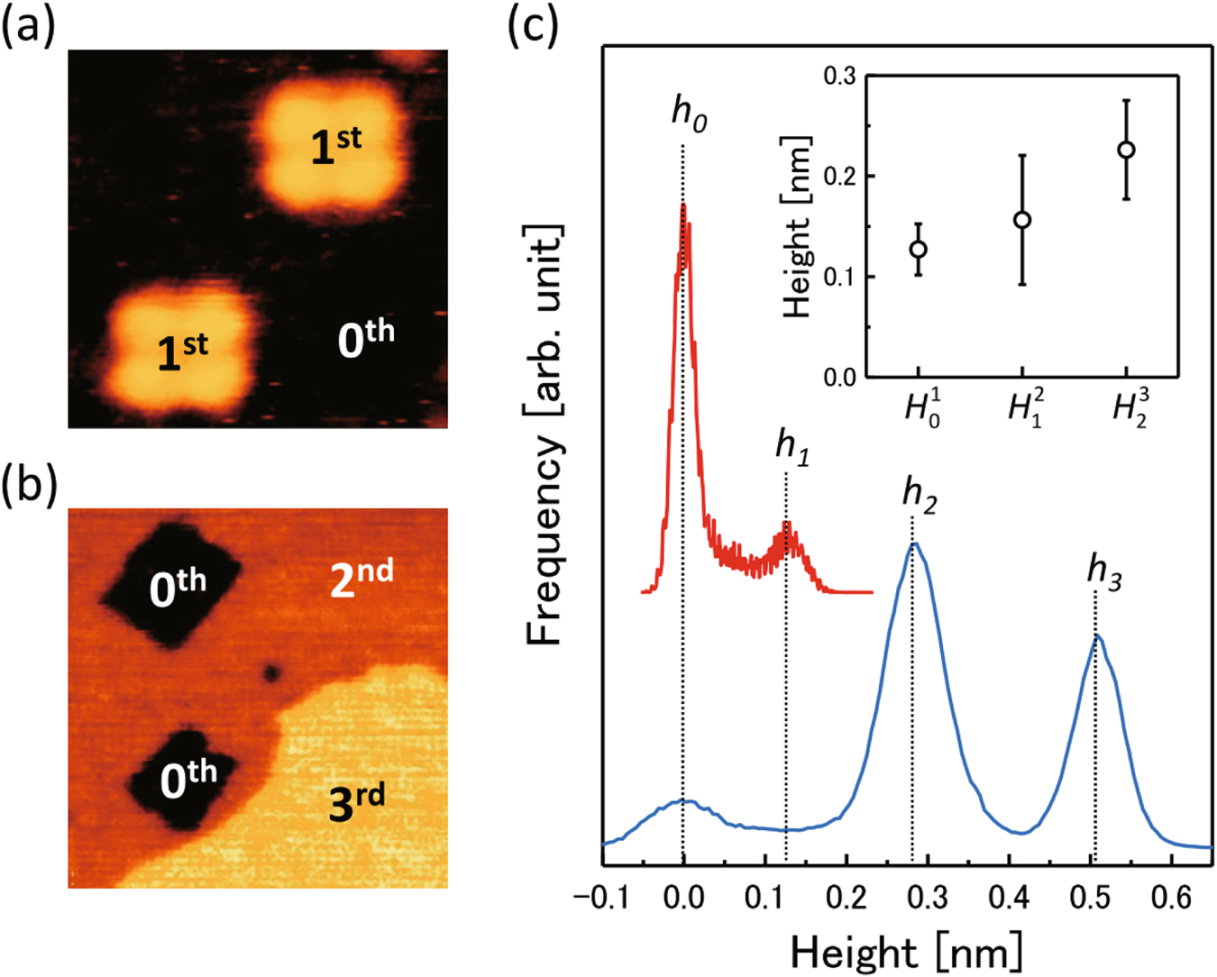
Topographic height distributions of H_2_Pc on Fe(001). (**a**,**b**) STM topography images of Fe(001) surfaces with (**a**) 0.1 ML (4.2 nm × 4.2 nm,–1.5 V, 300 pA), (**b**) 2.4 ML (28 nm × 28 nm, −1.5 V, 50 pA) coverage of H_2_Pc molecules. Labeled numbers, 0^th^, 1^st^, 2^nd^, and 3^rd^, indicate Fe(001) substrate, first, second, and third H_2_Pc layers, respectively. (**c**) Height histograms taken from (**a**) and (**b**), where height = 0 is defined as the topographic height of the Fe(001) substrate. Inset shows estimated layer distances (see text).

To obtain further insight into the formations of the second and third molecular films, the distributions of the pixel heights were analyzed from the STM images. In Fig. 4(a) and (b), we picked up two typical STM topography images; Fig. 4(a) includes the Fe(001) substrate and the first layer of the H_2_Pc single molecules, while Fig. 4(b) includes the Fe(001) substrate, the second layer, and the third layer. The apparent height distributions of the corresponding images are shown in Fig. 4(c). The remarkable peaks labeled *h*_0_, *h*_1_, *h*_2_, and *h*_3_ are attributed to the Fe(001) substrate, the first, second, and third layers of H_2_Pc, respectively. By fitting with the Gaussian functions, the peak positions and the widths (*h*_*i*_ ± *σ*_*i*_ for *i* = 0, 1, 2, 3) were determined. Then, the peak-to-peak intervals (${H}_{i-1}^{i}={h}_{i}-{h}_{i-1}$), which correspond to the interlayer distances between the *n*-th and the (*n* − 1)-th layers (0-th layer corresponds to the Fe(001) substrate), were evaluated. The results are shown in an inset of Fig. 4(c). ${H}_{i-1}^{i}$ increased monotonically with *i* (${H}_{0}^{1}=0.13\pm 0.03$ nm, ${H}_{1}^{2}=0.16\pm 0.06$ nm, and ${H}_{2}^{3}=0.23\pm 0.05$ nm), thereby indicating that the distance between the adsorbed molecules and their contacting substrate increases with layer number.

### Layer-dependent molecule–substrate interaction

We remark that the molecule–substrate distance is determined by the bonding strength. The theoretical calculation in our previous study^25^ demonstrated that the strong interaction between the *π*-conjugated H_2_Pc single molecule and the 3*d*-Fe state originates from the hybridized *π*–*d* bond. Owing to this strong interaction, the molecules are chemisorbed at the hollow sites of the Fe(001) surface with large adsorption energy (–4.2 eV) and the small molecular height from the substrate (0.15 nm). The feature is in good agreement with the present case, that is, the H_2_Pc single molecule stably exists on Fe(001) substrate with a small molecular height (${H}_{0}^{1}=0.13\pm 0.03$ nm). Our previous calculation also demonstrated that the molecular height becomes larger (0.2 nm) on Ag(001) where the molecules are physisorbed on the substrate with small adsorption energy (−1.5 eV). This physisorbed state allows the molecules to diffuse along the surface. In the present study, the second- and third-layer H_2_Pc molecules form well-packed films, which indicates that, at RT, the H_2_Pc single molecules thermally diffuse and stabilize by aggregations in individual layers. Therefore, the increase in interlayer distance [inset of Fig. 4(c)] is attributed to the weakened molecule–substrate interactions.

**Figure 5:**
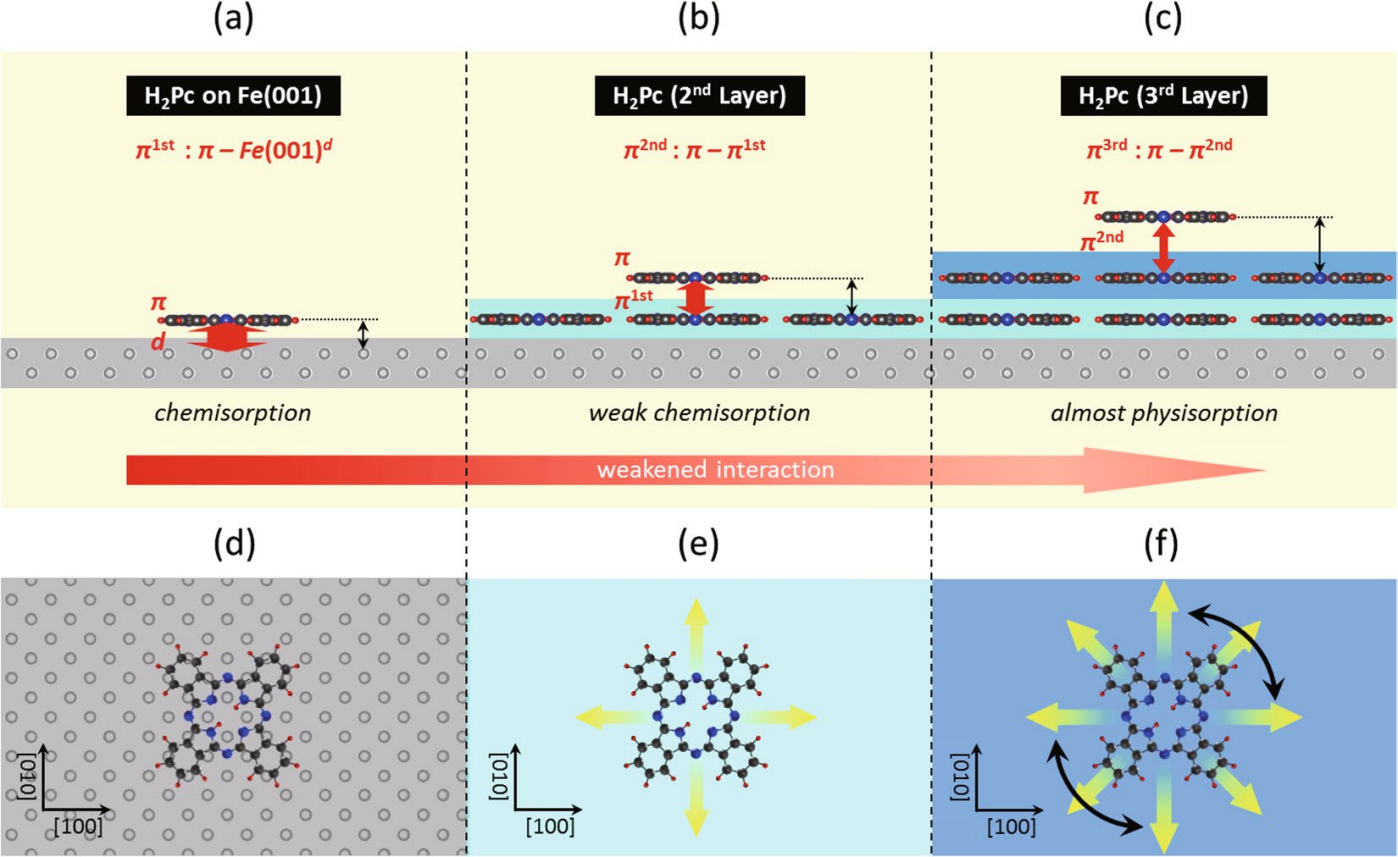
Layer-dependent H_2_Pc-substrate interactions. (**a**–**c**) Schematic illustration, representing dominant molecule–substrate interaction in first layer, (**a**), second layer, (**b**), and third layer, (**c**). (**d**–**f**) Schematic illustrations, representing diffusion of H_2_Pc single molecule in first layer, (**d**), second layer, (**e**), and third layer, (**f**).

Based on the result, we discuss the layer-dependent molecule–substrate interactions as illustrated schematically in Fig. 5(a)–(c). As previously mentioned, the chemical interaction of the first-layer molecule with the substrate attributes to the hybridized molecular *π* and Fe(001) *d*-states [Fig. 5(a)]. We suggest that this *π*–*d* state of the first-layer molecule (*π* ^1st^ state) interacts with the *π* state of the second-layer molecules, as sketched in Fig. 5(b). Due to the energy split of the hybridized *π*–*d* states^25^, *π* state of the second-layer molecule is energetically less resonant with *π* ^1st^ state. Thus, the *π*–*π* ^1st^ hybridization and the corresponding chemical bond should be weakened relative to those of the *π*–*d* states. By similar idea, the *π*–*π* ^1st^ state of the second layer molecule (*π* ^2nd^ state) should further interact with the *π* state of the third layer molecules [Fig. 5(c)]. Although such *π*–*π* ^2nd^ (=*π* ^3rd^) hybridization should be further weakened due to the energy split of the hybridized *π*–*π* ^1st^ state, the interaction should be different from pure van-der Waals type. This is because that, at RT, molecules physisorbed on substrate through *π*–*π* interactions do not from robust film^15^, while, in our experiment, the third-layer films were found to be stable [Fig. 3(c) and (d)]. The robust but rounded edge-shaped films indicate that some additional contributions other than van-der Waals interactions stabilized the films. Therefore, small but finite hybridization should be performed for *π*–*π* ^2nd^ states, and thus the third-layer molecules are considered to be in “almost-physisorbed” state [Fig. 5(c)].

It should be noted that the weakened hybridization enables the molecular diffusions in second and third layers, it still plays a significant role in the robust film formations at RT. Since such hybridization is triggered by the *π*–*d* state, the *d*-state of the magnetic metal is essentially important to stabilize the *π*-conjugated molecular films, and this is the crucial difference with the other noble metals. From a more common perspective, the role of *d*-state was further demonstrated by investigating stability of the other *π*-conjugated molecular film on a nonmagnetic metal substrate. As a system, metal-free tetraphenylporphine (H_2_TPP) on a Au(111) substrate (see Supplementary Method 2–3) was applied. It was found that the H_2_TPP molecules organized stable and 2D-ordered monomolecular films at 78 K, while the morphologies at RT become unstable (see Supplementary Figure 7 and Supplementary Note 2). It was revealed that such unstable film is due to the thermal diffusion of the physisorbed H_2_TPP molecules (see Supplementary Note 1), which attach/detach from the film edges with time. Therefore, *π*-conjugated molecular films cannot exist stably on nonmagnetic metals at RT, thereby further supporting the importance of the *d*-state in robust film formation.

### Layer-dependent film formation processes

Based on the layer-dependent molecule–substrate interaction, here we discuss the H_2_Pc film formation processes. On the bare Fe(001) substrate, the *π*–*d* chemisorbed bonds fix the adsorbed single molecules in particular in-plane configurations (type A and type B). This prevents molecular diffusion and aggregation even at RT [Fig. 5(d)], thereby leading to the disordered patchy structures, as shown in Fig. 1(g). On the first layer, the weakened chemical interaction allows for the molecular diffusion along the surface. However, due to the hybridized *π*–*π* ^1st^ states, the stable adsorption sites and the in-plane configurations of the molecules are considered to be still restricted by the Fe(001) substrate. As the molecular diffusion occurs by sequential hopping between the neighboring stable sites that are related to the Fe(001) surface, the second-layer molecule is considered to diffuse along the [100] and [010] directions [Fig. 5(e)]. We suggest that such restricted diffusion channel and/or the adsorbed configurations result in the aggregation and stabilization of the second-layer molecules along the [100] or [010] directions, forming rectangular shaped well-packed films [Fig. 2(a)]. One may argue that since the first layer forms disordered patchy structure, some of the second-layer molecules partially supported on the first layer should directly interact with the Fe(001) *d*-states through the vacant regions, which pins the molecules at the sites. Nevertheless, even in this case, the molecular diffusion should be allowed since such *π*–*d* interactions between separated molecule and Fe(001) substrate [${H}_{0}^{2}$~0.29 nm (see Fig. 4)] are considerably weakened from those of the first–layer molecules^25^. On the other hand, since the third–layer molecules are almost physisorbed on the second layer, they are considered to be in the several in-plane adsorbed configurations and allowed to diffuse two-dimensionally [Fig. 5(f)]. These features also result in the well-packed films but relatively rounded edges [Fig. 2(d)].

### Kinetic Monte Carlo simulation

**Figure 6:**
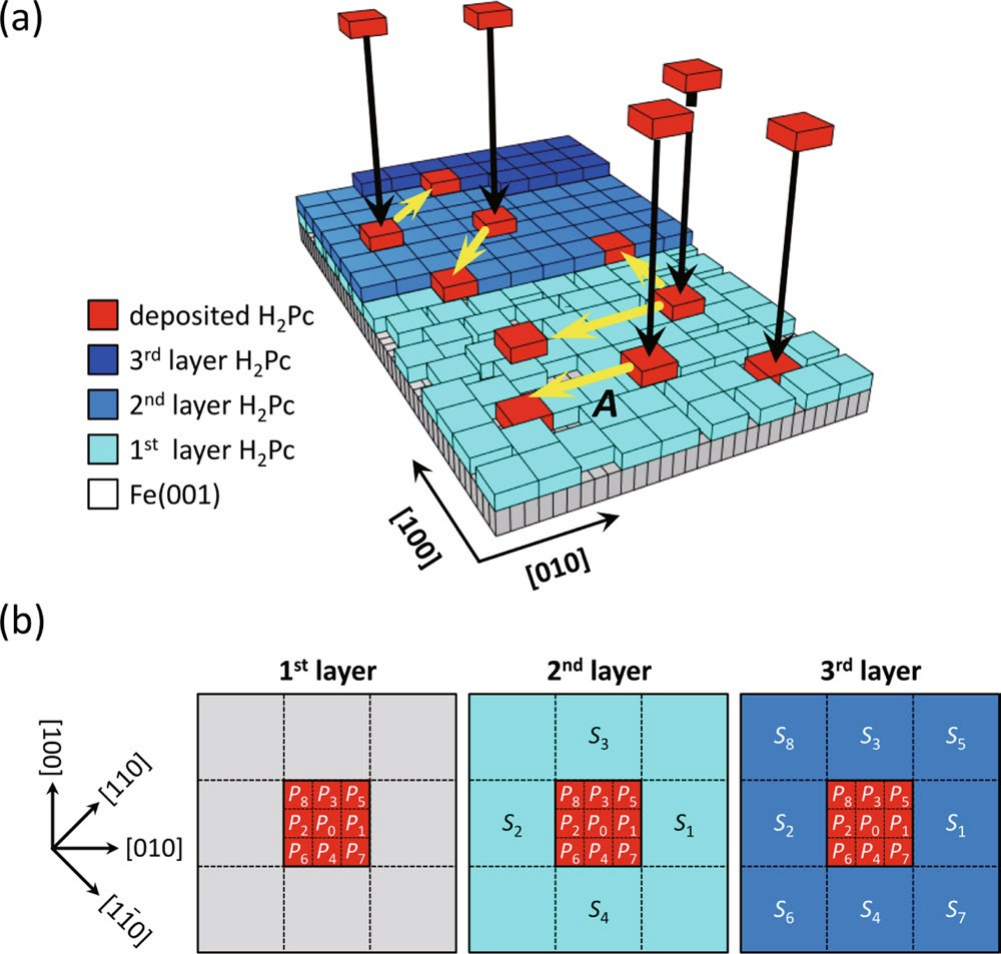
The method of the kinetic Monte Carlo simulation. (**a**) Schematic illustration representing deposition, diffusion and aggregation processes of H_2_Pc molecules on Fe(001) substrate. (**b**) Sketches of 9 × 9 grids square lattice including single adsorbed molecules (indicated by red rectangular area). *P*_*i*_(*i* = 0, 1, 2, …, 8) represent the hopping probabilities, and *S*_*i*_(*i* = 1, 2, 3, …, 8) represent the candidate sites where occupations of molecules freeze the target molecule.

To examine that the film growth processes we proposed in Fig. 5(d–f) actually result in the layer-dependent film morphologies, we performed kinetic Monte Carlo simulations and compared the resultant morphologies with the observed STM images. Figure 6(a) shows the scheme of our simulation, where depositions, diffusions, and aggregations of H_2_Pc molecules are included as elementary processes. The Fe(001) substrate was modeled as a 2D array of sites located along a 150 × 150 square lattice, which corresponds to a surface area of 43 nm × 43 nm (one lattice spacing corresponds to the bcc-Fe(001) lattice constant of 0.286 nm). Periodic boundary conditions were applied to the four edges of the 2D-grid to avoid discontinuity. For simplicity, this in-plane coordinate system (the 150 × 150 square lattice sites and the periodic boundary conditions) was also applied to the substrate surfaces of the second- and the third-layer molecules. H_2_Pc single molecules with a size of approximately 1.0 nm × 1.0 nm were modeled as 3 × 3 grids [red blocks in Fig. 6(a)], and deposited randomly with constant flux (50 molecules per one calculation loop) onto the substrate. Desorption of the deposited molecules from the substrate to vacuum was allowed with a probability *D*. Note that *D* value determines the total molecular coverage, but not the film morphologies (see Supplementary Figure 8). Therefore, the values were roughly set to reproduce the observed STM images, *D* = 0.1 for the first-layer molecules, *D* = 0.15 for the second-layer molecules, and *D* = 0.2 for the third-layer molecules (see Table 1), while we just considered that the *D* value increases with the layer number due to the weakened molecule–substrate interaction.

**Table 1:** Optimized parameters used in the Monte Carlo simulation. *D* is the desorption probability, *W*_*i* = 0, 1, 2, ..., 8_ the hopping weight, *T*_d_ the trapping weight, *ε* the potential depth of the Lennard-Jones potential, *σ* the distance at which the Lennard-Jones potential becomes zero, *N*_m_ the number of molecules required to freeze the target molecule (see text).

	*D*	*W*_0_	*W*_i_ _= 1, 2, 3, 4_	*W*_i_ _= 5, 6, 7, 8_	*T*_d_	*ε*	*σ*	*N*_m_
1^st^ layer H_2_Pc	0.1	arb. const.	0	0				
2^nd^ layer H_2_Pc	0.15	0	30	0	3	−30	3	2
3^rd^ layer H_2_Pc	0.2	0	50	50	3	−30	3	3

Molecular diffusion was set to be induced by sequential hopping of the molecules between the neighboring sites. To determine the hopping direction, we defined the hopping weight *W*_*i*_ and the corresponding hopping probability *P*_*i*_ as:

1 $${P}_{i}={W}_{i}/\sum _{j\mathrm{=0}}^{8}{W}_{j}(i=0,1,2,\mathrm{...},8),$$

where *P*_0_ corresponds to the probability that a molecule does not hop, *P*_*i*_ (*i* = 1, 2, 3, 4) the probabilities that a molecule hops along the [100] and [010] directions, and *P*_*i*_ (*i* = 5, 6, 7, 8) the probabilities that a molecule hops along the diagonal directions, that is, the [110] and [1 $\bar{1}$ 0] directions. The summary of *P*_*i*_ is shown in Fig. 6(b). The diffusion activation barrier, which was conventionally considered to determine *P*_*i*_^26,27^, was not considered. Alternatively, *P*_*i*_ was initialized and modified by fixing *W*_*i*_ under the following assumptions.

#### Layer-dependent effect

Based on the discussion in Fig. 5(d–f), the hopping directions were restricted depending on the layer number. Hopping of the molecules on the Fe(001) surface was prohibited. The molecules in the second layer were allowed to hop along the [100] and [010] directions. The molecules in the third layer were allowed to hop two-dimensionally. Analytically, *W*_*i*_ for the first, second, and third layers were fixed as

2 $${1}^{{\rm{st}}}{\rm{layer}}:{{\rm{W}}}_{i}=\{\begin{array}{ll}arbitrary\,constant\,(arb\mathrm{.}\,const\mathrm{.)} & (i=\mathrm{0)}\\ 0 & (i=1,2,3,\mathrm{...},\mathrm{8)},\end{array}$$

3 $${2}^{{\rm{nd}}}{\rm{layer}}:{W}_{i}=\{\begin{array}{ll}0 & (i=0,5,6,7,\mathrm{8)}\\ const\mathrm{.} & (i=1,2,3,\mathrm{4)},\end{array}$$

4 $${3}^{{\rm{rd}}}{\rm{layer}}:{W}_{i}=\{\begin{array}{ll}0 & (i=\mathrm{0)}\\ const\mathrm{.} & (i=\mathrm{1,}\,\mathrm{2,}\,\mathrm{3,}\,\mathrm{...,}\,\mathrm{8).}\end{array}$$

We can deduce that the *W*_*i*_ (*i* = 1, 2, 3, 4) for the second layer is smaller than *W*_*i*_ (*i* = 1, 2, 3, …, 8) for the third layer, since the molecules on higher layer are more mobile. However, more precise assignments of *W*_*i*_ require further information on the diffusion barrier which has not been obtained in the present experiments. Therefore, we tentatively assigned *W*_*i*_ (*i* = 1, 2, 3, 4) = 30 for the second-layer molecules and *W*_*i*_ (*i* = 1, 2, 3, …, 8) = 50 for the third-layer molecules (see Table 1), while we confirmed that simulations with different *W*_*i*_ values do not substantially change the results (see Supplementary Figure 9).

#### Intermolecular interaction

When more than two molecules are located within the same layer, *W*_*i*_ fixed previously was modified by the in-plane intermolecular interactions. Physically, this is equivalent to that the diffusion barrier is determined by the superposition between potential due to the molecule–substrate interaction and that due to the in-plane intermolecular interactions. For the interaction, the Lennard-Jones potential was applied. The potential was not treated quantitatively because we used the potential in order to determine the hopping weight qualitatively. Here, we assume that the *N* molecules, labeled *M*_*n*_ (*n* = 1, 2, 3, …, *N*), are located at the positions ***r***_*n*_ in the same layer. The force ***F***_*m*_(***r***_*m*_) acting on the target molecule *M*_*m*_ can be written as

5 $${{\boldsymbol{F}}}_{m}({{\boldsymbol{r}}}_{m})=-\sum _{n\ne m}\frac{\partial {U}_{mn}({r}_{mn})}{\partial {r}_{mn}}\frac{{{\boldsymbol{r}}}_{mn}}{{r}_{mn}},$$

with

6 $${U}_{mn}({r}_{mn})=4\varepsilon [(\sigma /{r}_{mn}{)}^{12}-{(\sigma /{r}_{mn})}^{6}\mathrm{].}$$

where ***r***_*mn*_ = ***r***_*n*_ − ***r***_*m*_, *r*_*mn*_ ≡ |***r***_*mn*_|, *U*_*mn*_ is the pairwise Lennard-Jones potential between *M*_*m*_ and *M*_*n*_, *ε* the potential depth, and *σ* the distance at which the intermolecular potential is zero. According to the direction and the magnitude of ***F***_*m*_, *W*_*i*_ of *M*_*m*_ was modified as follows. For the second-layer molecules, the hopping weights modified by the intermolecular interactions *W′*_*i*_ (*i* = 1, 2, 3, 4) were written as

7 $$\begin{array}{c}{W}_{1}^{\prime} =\{\begin{array}{ll}{W}_{1}+|{{\boldsymbol{F}}}_{m}\cdot {{\boldsymbol{e}}}_{{\rm{1}}}| & ({{\boldsymbol{F}}}_{m}\cdot {{\boldsymbol{e}}}_{{\rm{1}}} > \mathrm{0)}\\ {W}_{1} & ({{\boldsymbol{F}}}_{m}\cdot {{\boldsymbol{e}}}_{{\rm{1}}} < \mathrm{0)}\end{array}\end{array}$$

8 $${W}_{2}^{\prime} =\{\begin{array}{ll}{W}_{2} & ({{\boldsymbol{F}}}_{m}\cdot {{\boldsymbol{e}}}_{{\rm{1}}} > \mathrm{0)}\\ {W}_{2}+|{{\boldsymbol{F}}}_{m}\cdot {{\boldsymbol{e}}}_{{\rm{1}}}| & ({{\boldsymbol{F}}}_{m}\cdot {{\boldsymbol{e}}}_{{\rm{1}}} < \mathrm{0)}\end{array}$$

9 $${W}_{3}^{\prime} =\{\begin{array}{ll}{W}_{3}+|{{\boldsymbol{F}}}_{m}\cdot {{\boldsymbol{e}}}_{{\rm{2}}}| & ({{\boldsymbol{F}}}_{m}\cdot {{\boldsymbol{e}}}_{{\rm{2}}} > \mathrm{0)}\\ {W}_{3} & ({{\boldsymbol{F}}}_{m}\cdot {{\boldsymbol{e}}}_{{\rm{2}}} < \mathrm{0)}\end{array}$$

10 $${W}_{4}^{\prime} =\{\begin{array}{ll}{W}_{4} & ({{\boldsymbol{F}}}_{m}\cdot {{\boldsymbol{e}}}_{{\rm{2}}} > \mathrm{0)}\\ {W}_{4}+|{{\boldsymbol{F}}}_{m}\cdot {{\boldsymbol{e}}}_{{\rm{2}}}| & ({{\boldsymbol{F}}}_{m}\cdot {{\boldsymbol{e}}}_{{\rm{2}}} < \mathrm{0).}\end{array}$$

where ***e***_1_ and ***e***_2_ are the unit vectors along the [100] and [010] directions, respectively. For the third-layer molecules, *W′*_*i*_ (*i* = 1, 2, 3, 4) were written by Eqs (7)–(10). Additionally, *W′*_*i*_ (*i* = 5, 6, 7, 8) were written as

11 $${W}_{5}^{\prime} =\{\begin{array}{ll}{W}_{5}+|{{\boldsymbol{F}}}_{m}\cdot {{\boldsymbol{e}}}_{{\rm{3}}}| & ({{\boldsymbol{F}}}_{m}\cdot {{\boldsymbol{e}}}_{{\rm{3}}} > \mathrm{0)}\\ {W}_{5} & ({{\boldsymbol{F}}}_{m}\cdot {{\boldsymbol{e}}}_{{\rm{3}}} < \mathrm{0)}\end{array}$$

12 $${W}_{6}^{\prime} =\{\begin{array}{ll}{W}_{6} & ({{\boldsymbol{F}}}_{m}\cdot {{\boldsymbol{e}}}_{{\rm{3}}} > \mathrm{0)}\\ {W}_{6}+|{{\boldsymbol{F}}}_{m}\cdot {{\boldsymbol{e}}}_{{\rm{3}}}| & ({{\boldsymbol{F}}}_{m}\cdot {{\boldsymbol{e}}}_{{\rm{3}}} < \mathrm{0)}\end{array}$$

13 $${W}_{7}^{\prime} =\{\begin{array}{ll}{W}_{7}+|{{\boldsymbol{F}}}_{m}\cdot {{\boldsymbol{e}}}_{{\rm{4}}}| & ({{\boldsymbol{F}}}_{m}\cdot {{\boldsymbol{e}}}_{{\rm{4}}} > \mathrm{0)}\\ {W}_{7} & ({{\boldsymbol{F}}}_{m}\cdot {{\boldsymbol{e}}}_{{\rm{4}}} < \mathrm{0)}\end{array}$$

14 $${W}_{8}^{\prime} =\{\begin{array}{ll}{W}_{8} & ({{\boldsymbol{F}}}_{m}\cdot {{\boldsymbol{e}}}_{{\rm{4}}} > \mathrm{0)}\\ {W}_{8}+|{{\boldsymbol{F}}}_{m}\cdot {{\boldsymbol{e}}}_{{\rm{4}}}| & ({{\boldsymbol{F}}}_{m}\cdot {{\boldsymbol{e}}}_{{\rm{4}}} < \mathrm{0).}\end{array}$$

where ***e***_3_ and ***e***_4_ are the unit vectors along the [110] and [1 $\bar{1}$ 0] directions, respectively.

#### Trapping effect at the molecular vacancies

We considered the effect of vacant regions, which are formed primarily as sparse areas in the first layer. The second-layer molecule located at the molecular vacancy interacts with the Fe(001) *d*-state rather than the *π* ^1st^ state. Although this *π*–*d* interaction is weaker than that of the first-layer molecule owing to ${H}_{0}^{2} > {H}_{0}^{1}$ [see Fig. 4(c)], it can be considered stronger than the *π*–*π* ^1st^ interactions. Therefore, the molecule located at the molecular vacancy is more likely to stay at the site, thereby reducing the hopping probability. To include this effect in our simulation, a finite value *T*_d_ (=3 in the present case, see Table 1) was added to *W*_0_, that is, *P*_0_ becomes relatively increased while *P*_*i*_ (*i* = 1, 2, 3, …, 8) become relatively reduced. Note that this trapping effect should be considered only when the vacancy size is smaller than the single molecular size (3 × 3 grids). Otherwise (the vacancy size is larger than 3 × 3 grids), the molecule in the second layer moves to the first layer though the vacancy. This process is highlighted as *Process A* in Fig. 6(a).

#### Aggregation effect

We imposed an additional effect to stabilize the molecular hopping so that the molecules located adjacent to each other were set to become immobile. This aggregation effect was applied to the second- and third-layer molecules with different conditions, where two parameters were considered. One is the number of adjacent molecules *N*_m_ required to freeze the target molecule. Another is the candidates of the adjacent sites where occupations of molecules freeze the target molecule, which are labeled as *S*_*i*_ (*i* = 1, 2, 3, …, 8) in Fig. 6(b). As the diffusion and the in-plane adsorbed configurations of the second-layer molecules were restricted by the *π*–*π* ^1st^ interactions, the molecules were considered to be aggregated in particular directions, that is, the [100] and [010] directions. Therefore, the hopping of the second-layer molecule was set to be prohibited when not less than two molecules (*N*_m_ = 2, see Table 1) are located at either *S*_*i*_ (*i* = 1, 2, 3, 4) sites. In the third layer, in contrast, *π*–*π* ^2nd^ interactions allow the molecules to be physisorbed with several in-plane rotational angles and 2D diffusion with relatively higher mobility than the second-layer molecules. Correspondingly, their aggregation was also considered to be promoted two-dimensionally with larger *N*_m_ compared to the second-layer molecules. Therefore, the hopping of the third-layer molecule was set to be prohibited when not less than three molecules (*N*_m_ = 3, see Table 1) were located at either ${S}_{i}$ (*i* = 1, 2, 3, …, 8) sites.

After predetermined steps of calculations (including adsorptions, diffusions, and aggregations of all deposited molecules), the additional molecules were set to be re-deposited, and the calculation was performed in the same manner. This loop calculation was repeated until the total number of deposited molecules increases up to the predetermined coverages. The number of steps of the final calculation loop was set to be sufficiently large so that all the adsorbed molecules can stabilize sufficiently.

#### Resultant molecular morphologies

**Figure 7:**
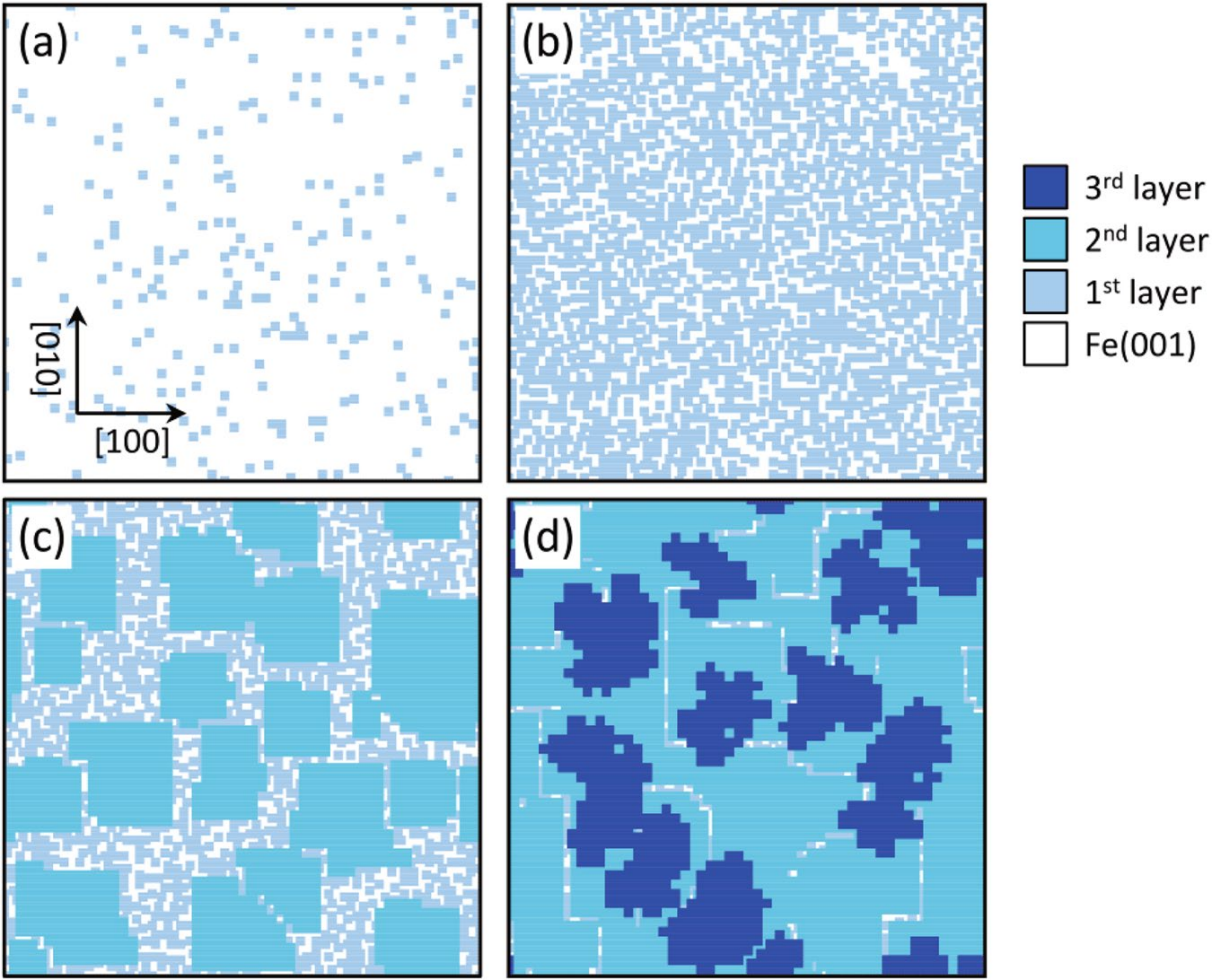
The result of the kinetic Monte Carlo simulation. H_2_Pc distributions on Fe(001) substrate simulated by the kinetic Monte Carlo method at different coverages, (**a**) 0.1 ML, (**b**) 0.8 ML, (**c**) 1.5 ML, (**d**) 2.4 ML.

Figure 7(a)–(d) shows the surface morphologies obtained by the simulations. The optimized parameters used in the simulations are summarized in Table 1. At a coverage of 0.1 ML [Fig. 7(a)], almost all molecules were isolated. With increasing coverage up to 0.8 ML [Fig. 7(b)], the surface was covered with the dispersed molecules. These results of the submonolayer coverages are in good agreement with the observed STM images [Fig. 1(f) and (g)]. Above monolayer coverages [Fig. 7(c) and (d)], the surface was covered with second and third H_2_Pc layers. They together formed densely packed structures, while the edge features were clearly different; the edges of the second layer aligned along the [100] and [010] directions, while those in the third layer were rounded. These features excellently reproduce the characteristic film morphologies [Fig. 2(a) and (d)].

In our film growth model, the second-layer molecules were assumed to diffuse along the [100] and [010] directions. However, one cannot completely exclude that the molecules diffuse also along diagonal direction with a small but finite probabilities since the present experiments could not quantitatively determine the strength of the *π*–*π* ^1st^ interactions. To test this case, we performed additional simulations where *W*_*i*_ _= 5, 6, 7, 8_ of the second-layer molecules were set to be small values (from 1 to 5). It should be note that such simulations also resulted in the rectangular shaped second-layer films (see Supplementary Figure 10). Thus, the characteristic film morphologies results not only from the simplistic assumptions. On the other hand, our simulation was simplified on the assumption that the molecules in second and third layers are adsorbed at 150 × 150 square lattice sites. This assumption slightly deviates from the STM image [Fig. 2(b)] where some molecules are relaxed to be adsorbed at the intermediate sites. Such relaxation disorders the molecular arrangement, while it will further promote the film compactness. Nevertheless, our simulation, at least, qualitatively well explains that the layer-dependent molecule–substrate interaction results in the characteristic film morphologies. The further experimental/theoretical studies on the diffusion barrier, adsorbed sites and configurations of film constituent molecules will enable the quantitative simulation that can more fully reproduce the film morphologies.

## Conclusion

We investigated the formation of *π*-conjugated H_2_Pc molecular films on a 3$d$-bcc-Fe(001) whisker surface at 300 K. On bare Fe(001), strong *π*–*d* chemisorbed bonds prevent molecular diffusion and aggregation, leading to disordered patchy structures, while densely packed films were formed in the second and third layers. The morphologies of such densely packed films were revealed to be stable even at 300 K. We also found that the second and third layer films show a clear difference in their morphologies. Partially p(5 × 5) ordered films with the rectangular edges were formed in the second layer, while the film edges in the third layer were rounded. From these finding, we suggested that the film growth processes and the resulting morphologies are still affected by the substrate bcc(001), and concluded that *d*-states of the magnetic Fe substrate play a crucial role in the robust film formation. Also, we discussed the layer-dependent molecule–substrate interaction and proposed the film growth model that was reasonably examined by the kinetic Monte Carlo simulation. The present results demonstrate that the monomolecular layers can be potentially utilized to precisely control the molecule–substrate interaction, which is a key for sophisticated molecular integration toward highly-functional devices.

## Methods

### UHV-STM system

The experiments were performed in a home-built UHV-STM system (base pressure <8 × 10^−9^ Pa). The system comprises a load-lock chamber, a sample preparation chamber, a molecular chamber, and a STM observation chamber. They are interconnected for performing substrate cleaning, molecular deposition, and STM observation without exposing the sample to the atmosphere.

### Sample preparation

An Fe whisker prepared by chemical vapor deposition method^28–30^ was introduced into the preparation chamber. The whisker was then cleaned and flattened by Ar^+^-ion sputtering (+0.8 kV, 30 min), with annealing the substrate at 870 K. The annealing was stopped 5 min after stopping the sputtering, which produced a clean and atomically flat Fe(001) surface^25^. Commercial H_2_Pc powder (Alfa Aesar, purigy 95 %) was purified by sublimation at 653 K and recrystallization at 473 K under a pressure of 10^−3^ Pa (yield 30 %). The obtained high-purity H_2_Pc powder was then located into a crucible, which was fixed in the molecular chamber, and it was first degassed at a temperature of 550 K for 5 min. During degasification, the valve between the molecular chamber and the sample preparation chamber was closed. After degassing, the H_2_Pc molecules were deposited on the Fe(001) substrate by opening the valve with the crucible held at 550 K. During deposition, the Fe(001) substrate was held at RT. Moreover, annealing was not performed after deposition. The deposition rate was controlled at a value of approximately 0.11 monolayer (ML)/min.

### STM measurements

The STM measurements were performed in the constant current mode using electrochemically etched W-tips (*ϕ* = 0.3 mm, purity 99.99 %) that were carefully cleaned by annealing in the load-lock chamber prior to utilization^31^. Bias voltage (*V*_s_) was applied to the sample with respect to the tip being held at the virtual ground potential. Tunneling current was detected from the tip. All STM images was obtained at 300 K. Lateral (in-plane) thermal drift in our STM system is around 0.02 nm/s. Only one drift-induced distortion image (see Supplementary Figure 1) was analytically corrected [Fig. 2(b)] by applying an inverse distortion transformation^32^.

## Electronic supplementary material

supplementary information
