# Supplementary material for: Room temperature stable film formation of π-conjugated organic molecules on 3d magnetic substrate
Source: Sci Rep. 2018 Jan 10;8:353. doi: 10.1038/s41598-017-18605-2 (PMC5762783; doi:10.1038/s41598-017-18605-2)
Supplement: Supplementary file 1 — supplementary information [file 41598_2017_18605_MOESM1_ESM.pdf]

Supplementary Information

Room temperature stable film formation of *π*-conjugated organic molecules on 3*d* magnetic substrate

Eiichi Inami[1,*], Mikio Shimasaki[2], Hideki Yorimitsu[3] & Toyo Kazu Yamada[1,4,†]

[1] Graduate School of Science and Engineering, Chiba University,
1-33 Yayoi-cho, Inage-ku, Chiba 263-8522, Japan,
[2] Graduate School of Advanced Integration Science, Chiba University,
1-33 Yayoi-cho, Inage-ku, Chiba 263-8522, Japan,
[3] Department of Chemistry, Graduate School of Science, Kyoto University,
Kitashirakawa, Oiwakecho, Sakyo-ku, Kyoto 606-8502, Japan,
[4] Molecular Chirality Research Center, Chiba University,
1-33 Yayoi-cho, Inage-ku, Chiba 263-8522, Japan,

[*] e_inami@chiba-u.jp
[†] toyoyamada@faculty.chiba-u.jp

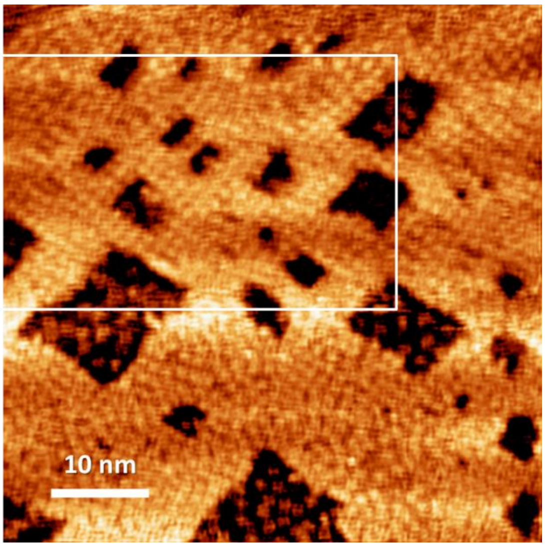

Supplementary Figure 1. H_2Pc film on Fe(001). STM topography image (55 nm × 55 nm, V_s= –2.0 V, I_t = 500 pA) of H_2Pc film on Fe(001) without drift compensation. To reduce the image distortion due to the thermal drift, the rectangular region was extracted and then stretched vertically, obtaining the image of Fig. 2(b) in the main manuscript.

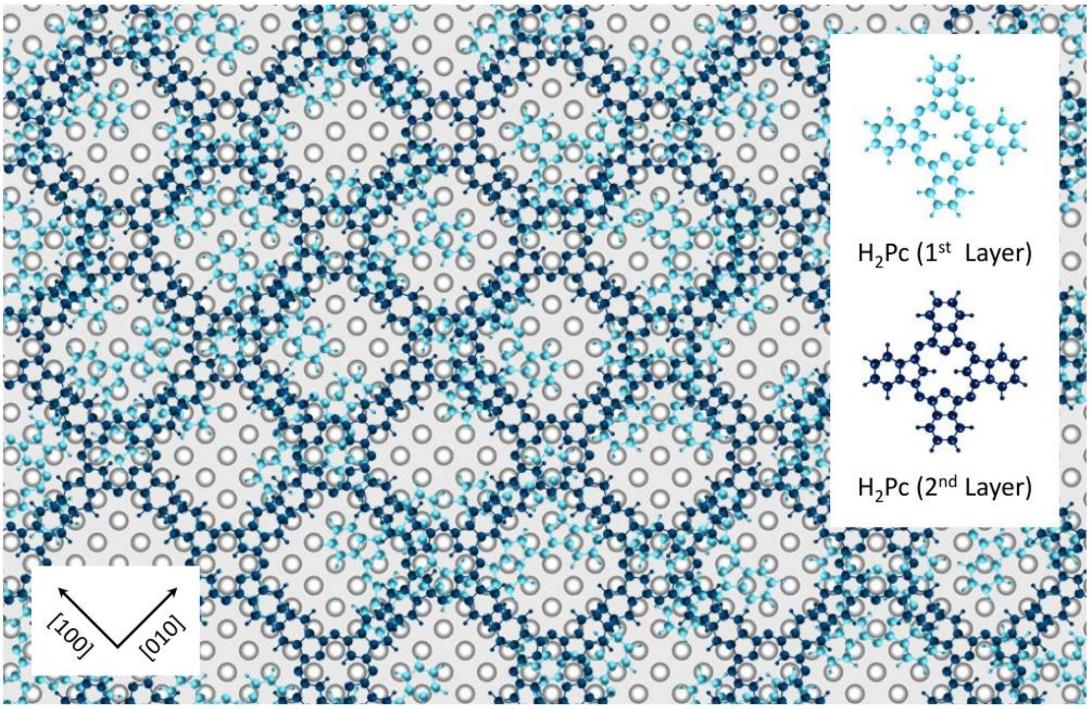

Supplementary Figure 2. Sketch of the proposed molecular arrangement of H_2Pc film. The model was proposed under the assumption that the second-layer molecules are adsorbed with type A configuration (see main manuscript). This model is not energetically favorable due to the repulsive H-H interactions between benzene groups of neighboring molecules. The model with type B configuration (see main manuscript), which is energetically favorable, is shown in the main manuscript.

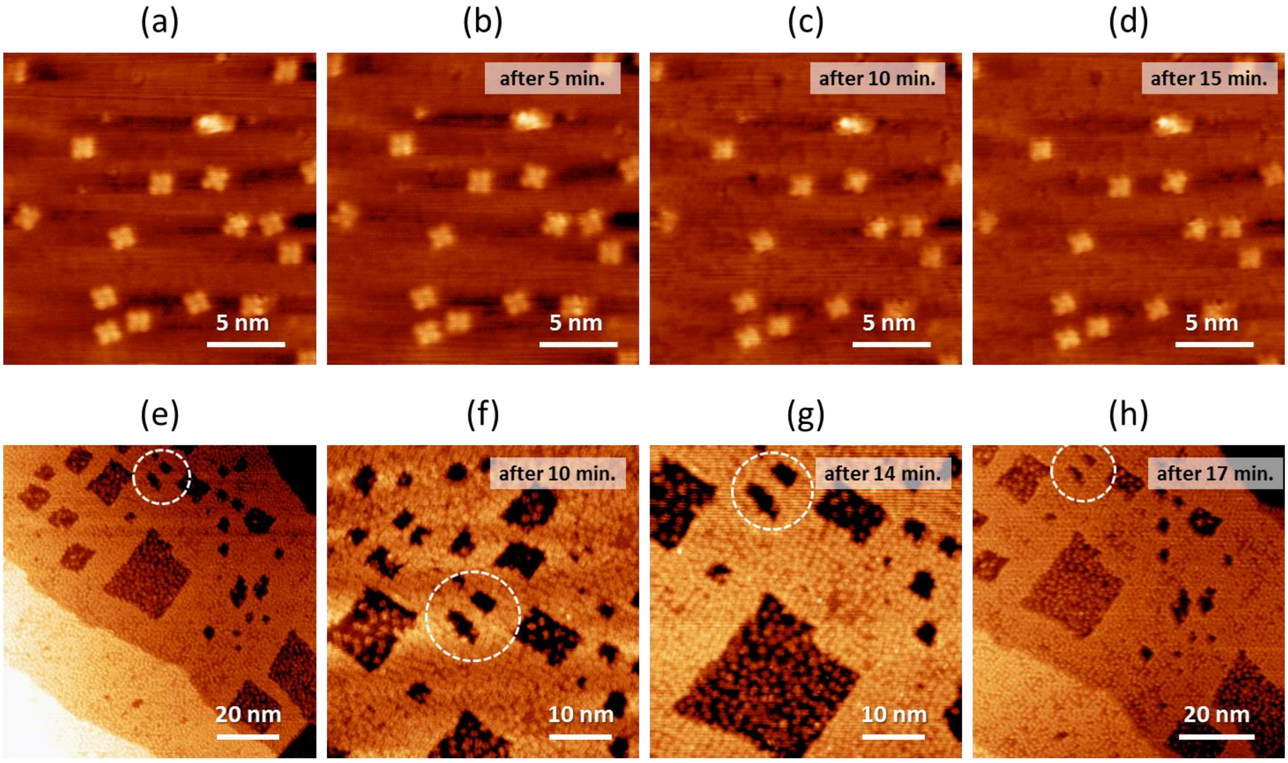

Supplementary Figure 3. Sequential STM images of H_2Pc molecules on Fe(001). (a)-(d) Sequential STM image of H_2Pc single molecules on Fe(001) (20 nm × 20 nm, V_s= -1.0 V, I_t = 200 pA). (e)-(g) Sequential STM image of H_2Pc molecular films on Fe(001) (V_s= -2.0 V, I_t = 200 pA). The size of each image is 100 nm × 100 nm, (e), 50 nm × 50 nm, (f)-(g), 83 nm × 83 nm, (h). The dashed circles in (e)-(h) mark the identical area.

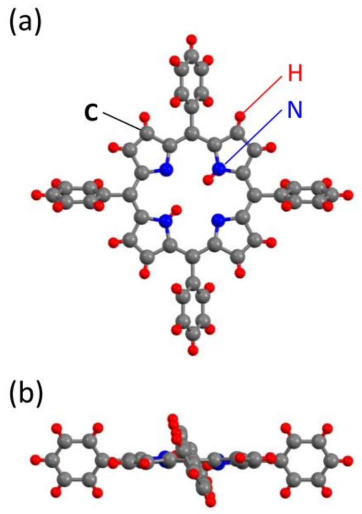

Supplementary Figure 4. Structural ball-stick model of the H_2TPP molecule. (a) top view, (b) side view.

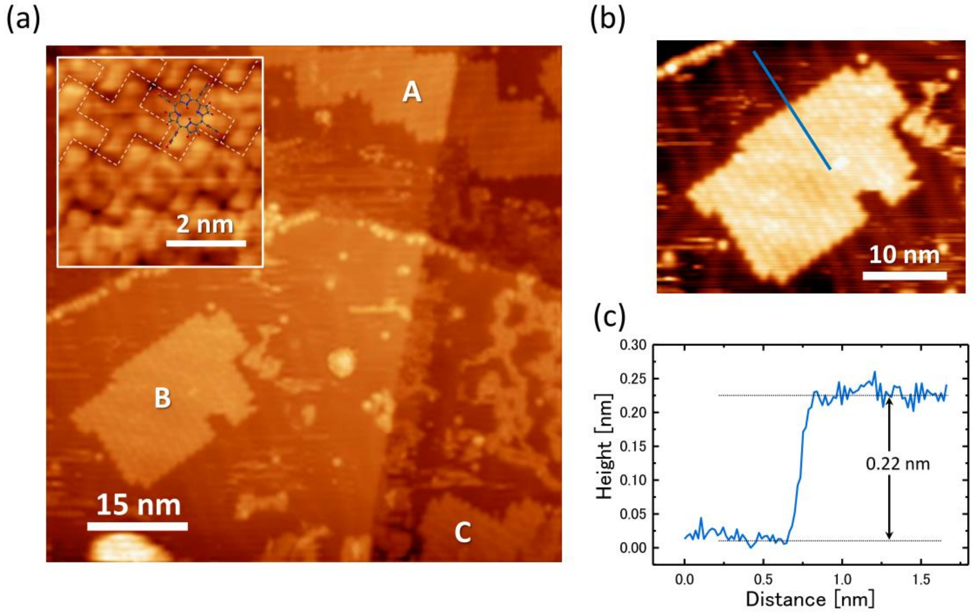

Supplementary Figure 5. STM images of H_2TPP films on Au(111) surface. (a) STM topography image of H_2TPP monolayer films on Au(111) surface at 78 K (75 nm × 75 nm, V_s = –1.4 V, I_t = 30 pA). Molecular films, labeled *A*, *B*, and *C*, are in three-fold symmetry. The inset shows the expanded scale image of film *A*. (b) Expanded scale image of film *B*. (c) Topographic height profile along the blue line in (b).

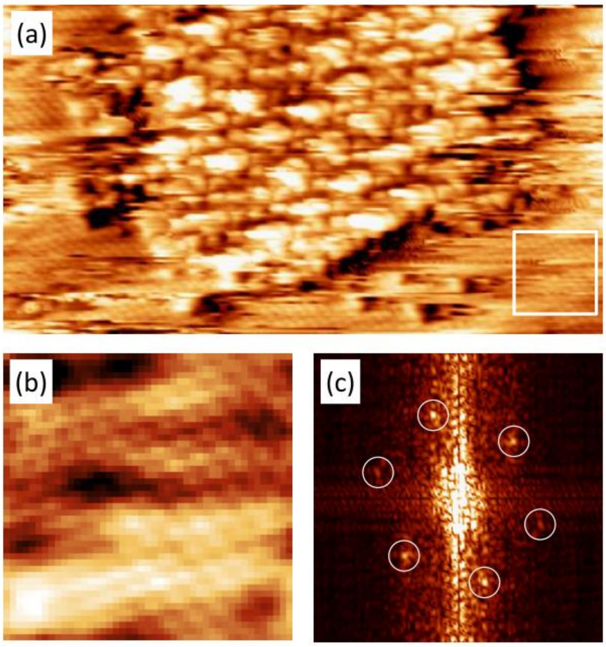

Supplementary Figure 6. Atom-resolved STM images of H_2TPP film on Au(111) surface. (a) STM topography image of H_2TPP monolayer films on Au(111) surface acquired at 78 K (20 nm × 10 nm, V_s = –1.3 V, I_t = 77.5 pA). (b) Expanded scale image of the rectangular area in (a), showing the atomic arrangement of Au(111) surface. (c) 2D fast Fourier transformed image of (b). Bright spots surrounded by circles attribute to the hexagonal lattice of the Au(111) substrate.

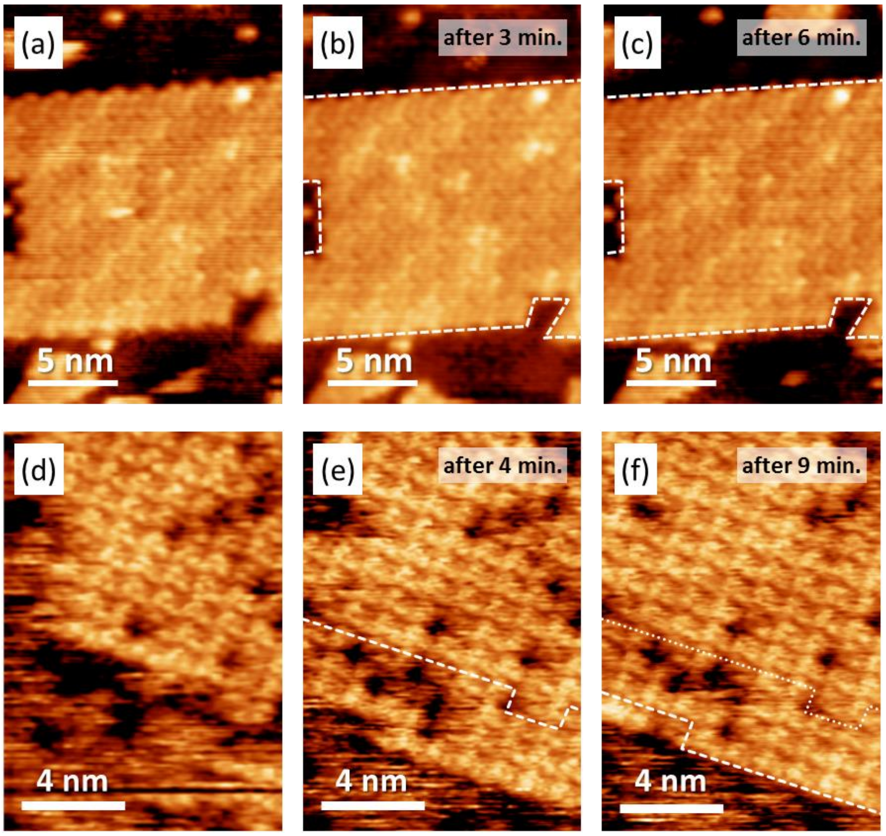

Supplementary Figure 7. Continuous STM images of H_2TPP films on Au(111). (a)-(c) were a set of continuous images acquired at 78 K (22 nm × 15 nm, V_s= –1.4 V, I_t = 130 pA), while (d)-(f) were the other set of images acquired at 300 K (15 nm × 11 nm, V_s = –1.3 V, I_t = 40 pA).

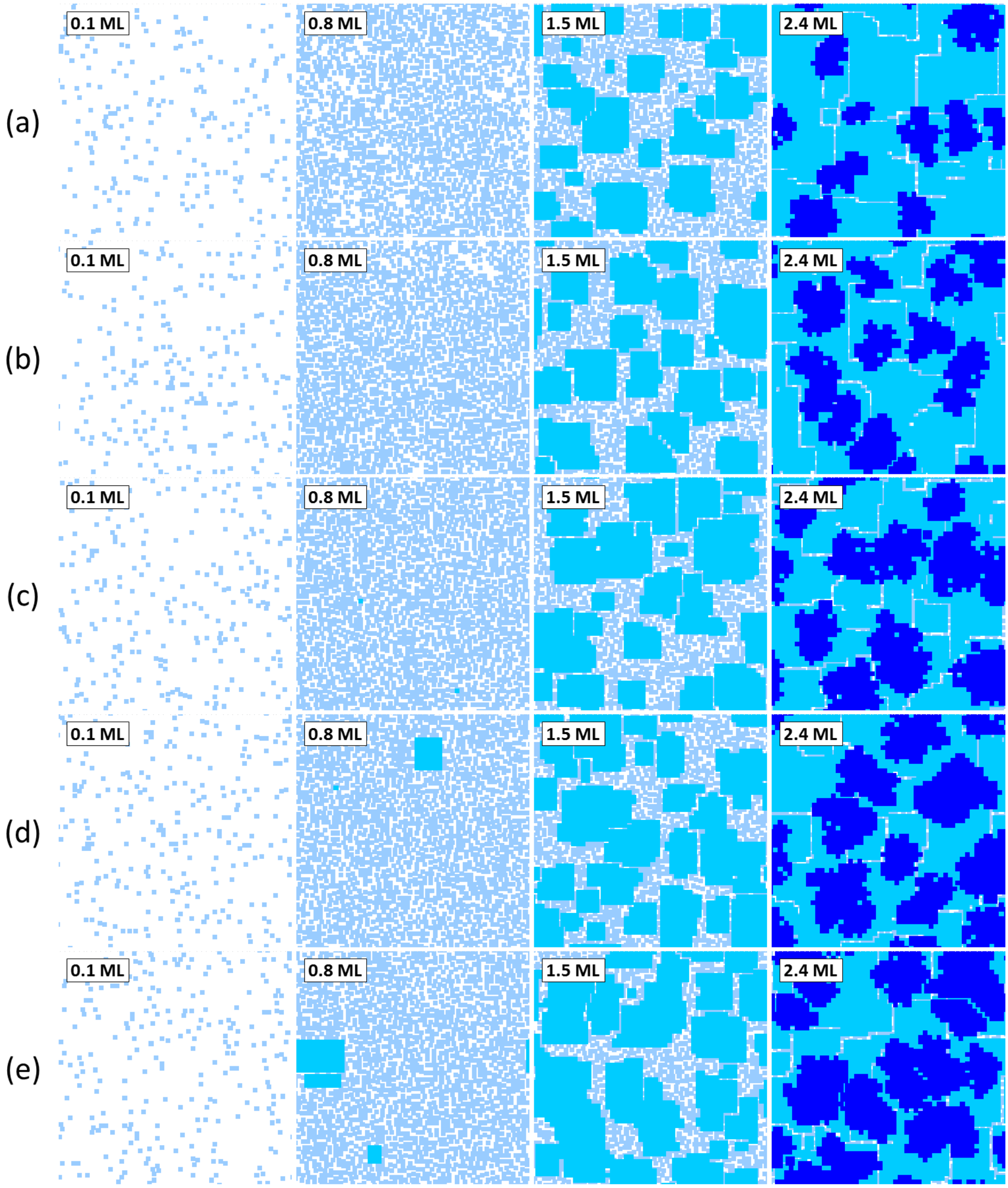

Supplementary Figure 8. H_2Pc distributions on Fe(001) simulated by kinetic Monte Carlo method. Desorption probability *D* was set as follows: (a) D_i (1st) = 15 %, D_i (2nd) = 20 %, D_i (3rd) = 25 %. (b) D_i (1st) = 10 %, D_i (2nd) = 15 %, D_i (3rd) = 20 %, (c) D_i (1st) = 7 %, D_i (2nd) = 12 %, D_i (3rd) = 17 %, (d) D_i (1st) = 5 %, D_i (2nd) = 10 %, D_i (3rd) = 15 %, (e) D_i (1st) = 2 %, D_i (2nd) = 7 %, D_i (3rd) = 12 %. The other parameters were set to be the same values as in Table I in Main manuscript.

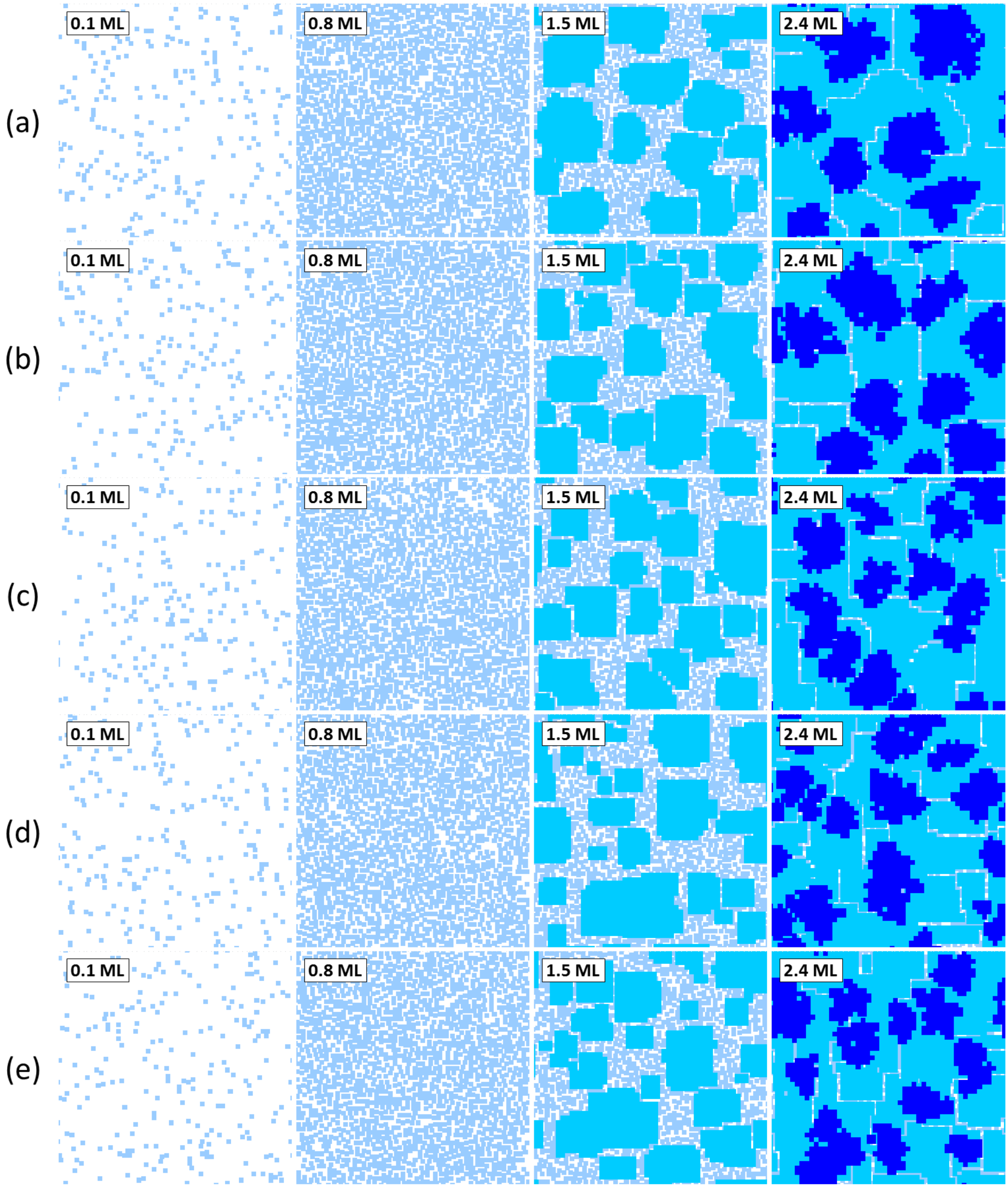

Supplementary Figure 9. H_2Pc distributions on Fe(001) simulated by kinetic Monte Carlo method. Hopping rate of the second layer molecules $W_{i\,=\,1,2,3,4}$ were set as 10, (a), 20, (b), 30, (c), 40, (d), 50, (e). Hopping rate of the third layer molecules $W_{i\,=\,1,2,3,\ldots,8}$ were set as 30, (a), 40, (b), 50, (c), 60, (d), 70, (e). The other parameters were set to be the same values as in Table I in Main manuscript.

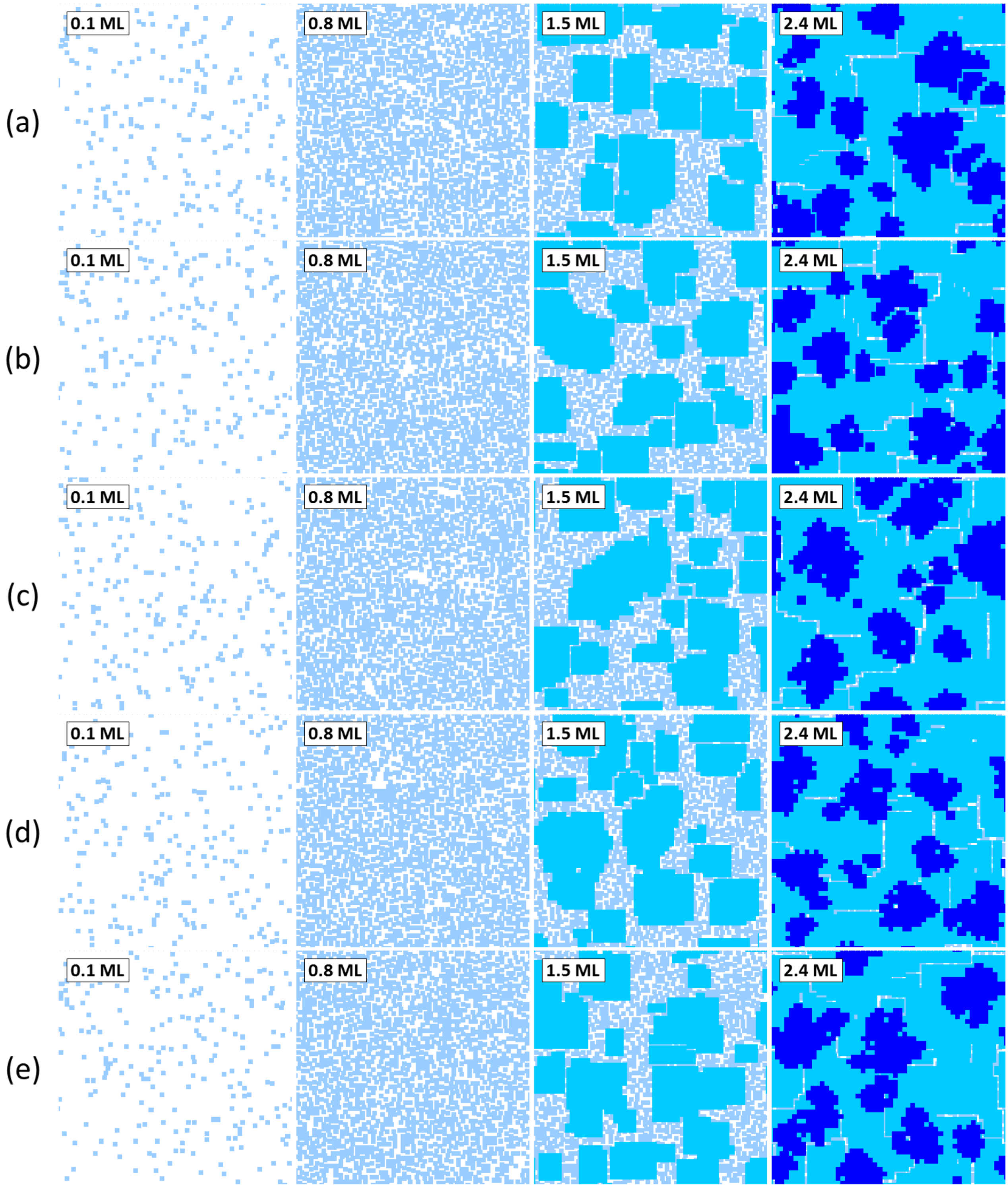

Supplementary Figure 10. H_2Pc distributions on Fe(001) simulated by kinetic Monte Carlo method. Hopping rate of the second layer molecules $W_{i\,=\,1,2,3,4}$ and the third layer molecules $W_{i\,=\,1,2,3,\ldots,8}$ were set as 30 and 50, respectively. $W_{i\,=\,5,6,7,8}$ of the second layer molecules were set as 1, (a), 2, (b), 3, (c), 4, (d), 5, (e). The other parameters were set to be the same values as in Table I in Main manuscript.

Supplementary Method 1: Extracting Consecutive STM images at the same area

Due to the lateral thermal drift, the consecutive STM imaging results in the shift of the imaging area. To address this issue, we carried out the following procedures. First, during the STM imaging, we estimated roughly the directions of the lateral thermal drift, that is, we checked which directions the image area shifts at every imaging. Based on the obtained information on the lateral drift direction, we could intentionally change the imaging area to reduce the effect of the image shift. By this procedure, the particular morphology of the single molecules or the molecular films can be traced (see Supplementary Figure 3). Second, based on the obtained STM images, the overlap regions (indicated by the rectangular areas in Supplementary Figure 3) have been analytically evaluated, and then, extracted from the images. The extracted areas of initial images [Supplementary Figure 3(a) and (e)] and final images [Supplementary Figure 3(d) and (h)] were highlighted in Fig. 3 of the main manuscript.

Supplementary Method 2: Preparation of H_2TPP film on Au(111)

A single crystalline Au(111) substrate was subjected to several cycles of Ne^+-ion sputtering (+1.0 kV, 10 min) and subsequent annealing (800 K, 10 min) in the sample preparation chamber, by which cleaned and atomically-flat surface was obtained. The H_2TPP powder (see Supplementary Figure 4) located in a crucible was first degassed at 443 K for 5 min, and then, deposited onto the clean Au(111) surface. During the deposition process, the temperature of the crucible and the Au(111) substrate was maintained at 443 K and at 300 K, respectively. No annealing of the substrate was performed after deposition.

Supplementary Method 3: STM measurements

The H_2TPP film on Au(111) was characterized by STM measurements at 78 K and 300 K. The imaging was performed in the constant current mode. Electrochemically etched W-tips (ϕ = 0.3 nm, purity 99.99 %) were carefully cleaned up by annealing in the load-lock chamber prior to use. Bias voltage was applied to the sample with respect to the tip being held at virtual ground potential. Tunneling current was detected from the tip.

Supplementary Note 1: STM characterization of H_2TPP film on Au(111) at 78 K

A low-temperature (78 K) STM measurement first confirmed that the H_2TPP molecules organize 2D ordered square lattice on bare Au(111) surface where the herringbone reconstruction was clearly resolved [see Supplementary Figure 5(b)]. The H_2TPP monolayer films were rotated by 120 deg. with respect to each other owing to the threefold symmetry of the Au(111) surface [see Supplementary Figure 5(a)]. The matrix notation of the molecular superstructure was obtained as

$$\begin{pmatrix} 6 & -2 \\ -2 & 6 \end{pmatrix}$$

with respect to the Au(111) unit cell (see Supplementary Figure 6). The image analysis also revealed that the H_2TPP monolayer height from the Au(111) surface was approximately 0.22 nm [see Supplementary Figure 5(c)], which is almost equal to the case of the H_2Pc/Ag(001) system [1]. These features indicate that the H_2TPP molecules are physisorbed on the Au(111) substrate, which enables diffusion and the following film formations.

Supplementary Note 2: Thermal stability of H_2TPP film on Au(111) substrate

To check the stability of the H_2TPP monolayer film on Au(111) substrate, we performed the continuous STM imaging, in the same manner as that of H_2Pc film (Fig. 3 in the body of the manuscript or Supplementary Method 1). Supplementary Figure 7 shows the sequential STM images of the H_2TPP monolayer film on the Au(111) substrate acquired at 78 K, (a)-(c), and 300 K, (d)-(f), respectively. At 78 K, the H_2TPP film maintained the molecular arrangement and the edge feature. At 300 K, however, the edge changed remarkably with time. Furthermore, we observed considerable noise at the Au(111) terrace, which is an indication for interactions between the tip and the moving molecules [2]. This result clearly indicate that the mobility of the molecule on this surface is enough large at RT, where adsorption and desorption of the molecules at the film edges, i.e. film growths and collapses, are steadily induced. These results clearly indicate that the H_2TPP film cannot exist stably on Au(111) surface at RT.

Supplementary References

1. Yamada, T. K., Yamagishi, Y., Nakashima, S., Kitaoka, Y. & Nakamura, K. Role of *π-d* hybridization in 300-K organic-magnetic interface: metal-free phthalocyanine single molecules on bcc Fe(001)-whisker. *Phys. Rev. B* **94**, 195437 (2016).
2. Naitoh, Y., Matsumoto, T., Sugiura, K., Sakata, Y. & Kawai, T. Self-assembled stripe structure of zinc–phthalocyanine on graphite surfaces. *Surf. Sci.* **487**, L534–L540 (2001).
